# Rethinking the basic-applied dichotomy

**DOI:** 10.1186/s41235-016-0011-x

**Published:** 2016-09-22

**Authors:** Jeremy M. Wolfe

**Affiliations:** 1 Departments of Ophthalmology & Radiology, Harvard Medical School, 64 Sidney St. Suite. 170, Cambridge, MA 02139-4170 UK; 2 Visual Attention Lab Department of Surgery, Brigham & Women’s Hospital, 64 Sidney St. Suite. 170, Cambridge, MA 02139-4170 UK

## Editor in Chief: *Cognitive Research: Principles and Implications*

*Cognitive Research: Principles and Implications* (CRPI) is a new journal with ambitions. Not only do we want to publish first-rate cognitive research, we want to change the standard way that our discipline thinks about basic and applied research. The standard view is dichotomous: Is your research basic, fundamental, and pure or applied, translational, and practical? We know about dichotomies in our trade. Remember when you learned about the nature-nurture debate? The topic might have been intelligence or mental illness, and the question was framed in terms of whether this attribute of our lives was due to our genetic endowment or to what we had learned in the world. Some cases were reasonably clear: eye color — nature; memory for the names of U.S. Republican presidential candidates — nurture. However, for the more interesting cases, we learned soon enough that the correct answer is almost never nature *or* nurture; it is *both*. Having gotten that question right on the midterm, we moved on to a discussion of how much of intelligence or mental illness or whatever can be attributed to genes and how much to environment. Eye color and Republican candidates’ names hold down the two ends of a nature-nurture continuum, but where is something like intelligence on that continuum? The press and the Internet are fond of this exercise. “Thank your parents if you are smart,” says the United Kingdom’s *Daily Mail*, because “up to 40 % of a child’s intelligence is inherited.” What does such a claim mean? The article does go on to say that this is an estimate of how much variance is explained by genetics. Interestingly, these discussions of apportioning variance tend to stick with the main effects and never discuss the interaction term in this implicit two-way analysis of variance, though much of the action presumably lies in that interaction term.

**Fig. 1:**
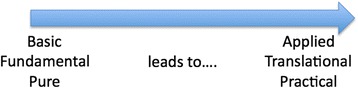
The “standard model” of basic and applied research

So, is your research “basic” or “applied”? It is possible that your latest paper describes work that could be labeled as one or the other, but, as in nature vs nurture, it is probably neither purely one nor purely the other. That leads us to imagine a continuum between basic and applied poles. In the common view (and, perhaps, in your last grant proposal), basic and applied research not only lie on a line but also define a vector with a direction. Today’s basic research will lead to tomorrow’s application (Fig. 1).

**Fig. 2:**
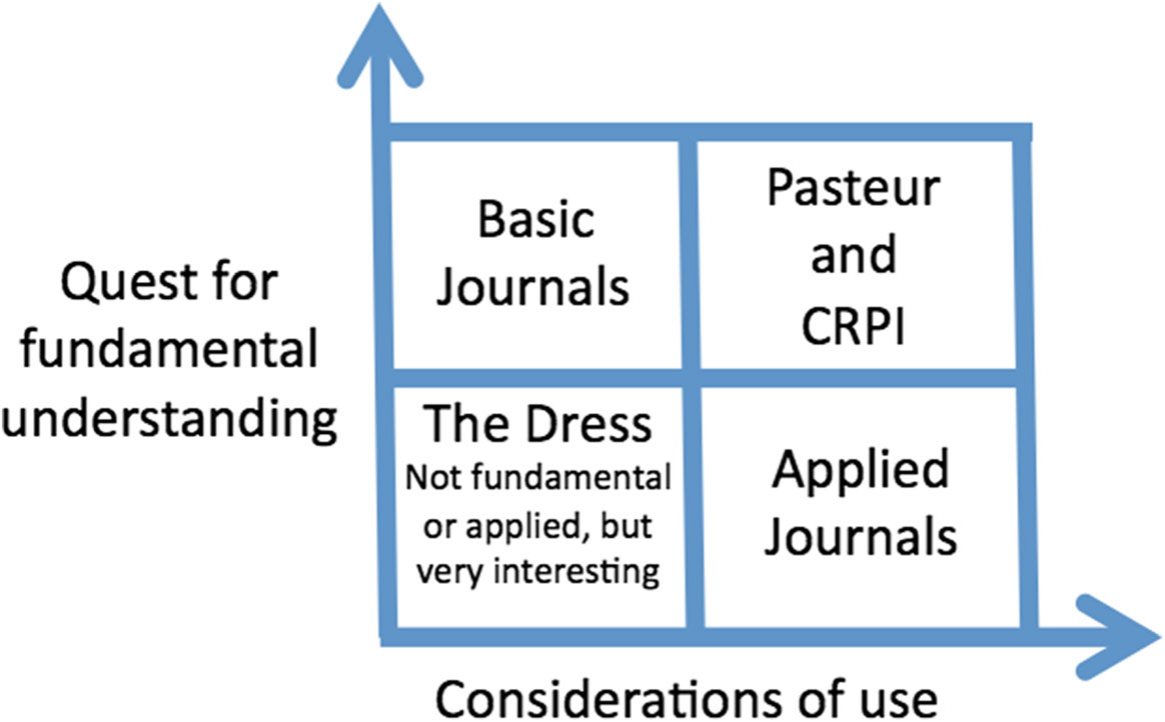
A modified version of Stokes’s quadrants. The “dress” in the lower left corner refers to the Internet frenzy over the dress that looked blue and black to some, white and gold to others. See https://en.wikipedia.org/wiki/the_dress_(viral_phenomenon). *CRPI Cognitive Research: Principles and Implications*

At CRPI, we are inspired by Donald Stokes’s 1997 book *Pasteur’s Quadrant: Basic Science and Technological Innovation* to look differently at our scientific enterprise. His favorite example, as given in the title, is Louis Pasteur (think pasteurized milk). Pasteur’s work in microbiology, Stokes argues, makes both basic and applied contributions. That would seem to place his work either at both ends of the continuum at the same time or in the middle, but the middle could also be the location of research that makes *neither* a basic *nor* an applied contribution. Stokes’s solution is to envision a two-dimensional, not a one-dimensional, research space. On his *x*-axis, we have “considerations of use,” and on the *y*-axis, we have the “quest for fundamental understanding.” If we imagine this as a 2 × 2 grid, that upper right quadrant is the “Pasteur’s quadrant” of his title and the sweet spot for submissions to CRPI (Fig. 2).

Stokes’s two-dimensional view is also intended to disrupt the idea of a one-way street from basic to applied work. Science is a dialogue between the world and the laboratory. Work on semiconducting materials was basic research before the invention of the transistor, but it might be considered “applied” afterward (Brooks, 1979). Our research is part of a similar dialogue. What is the dose-response curve relating light exposure shifts in the circadian clock (Boivin, Duffy, Kronauer, & Czeisler, 1996)? That is a fundamental question about our sleep-wake cycle that might not have arisen if we had not invented jet lag. In my own research life, I would not have asked the basic question of how target prevalence influences visual search if I had not noticed that airport baggage screeners were searching for a target that was almost never there (Wolfe, Horowitz, & Kenner, 2005). This is what Stokes calls “use-inspired, basic research,” and use-inspired, basic research is CRPI’s bread and butter.

At CRPI, we respect the work in any part of Stokes’s two-dimensional space. Our specific mission is to encourage and advertise the two-way conversation between the world and the cognitive laboratory. The aim of CRPI is to be a scholarly journal filled with the best of cognitive science research. At the same time, if someone from outside our field looks at our abstracts and, even more so, at our significance statements, they should see “research in an area of basic scientific ignorance that lies at the heart of a social problem” (Stokes, 1997, p. 60, quoting Holton, 1993, p. 115). We are firmly convinced that Pasteur’s quadrant holds much of the best cognitive research, and we want to publish papers in that area.
